# DDR1 and Its Ligand, Collagen IV, Are Involved in In Vitro Oligodendrocyte Maturation

**DOI:** 10.3390/ijms24021742

**Published:** 2023-01-16

**Authors:** Maria Elena Silva, Matías Hernández-Andrade, Nerea Abasolo, Cristóbal Espinoza-Cruells, Josselyne B. Mansilla, Carolina R. Reyes, Selena Aranda, Yaiza Esteban, Ricardo Rodriguez-Calvo, Lourdes Martorell, Gerard Muntané, Francisco J. Rivera, Elisabet Vilella

**Affiliations:** 1 Laboratory of Stem Cells and Neuroregeneration, Institute of Anatomy, Histology and Pathology, Faculty of Medicine, Universidad Austral de Chile, Valdivia 5090000, Chile; 2 Center for Interdisciplinary Studies on the Nervous System (CISNe), Universidad Austral de Chile, Valdivia 5090000, Chile; 3 Institute of Pharmacy, Faculty of Sciences, Universidad Austral de Chile, Valdivia 5090000, Chile; 4 Hospital Universitari Institut Pere Mata, Institut d’Investigació Sanitària Pere Virgili-CERCA, Universitat Rovira i Virgili, 43206 Reus, Spain; 5 Centro de Investigación Biomédica en Red en Salud Mental, CIBERSAM-Instituto de Salud Carlos III, 28029 Madrid, Spain; 6 Vascular Medicine and Metabolism Unit, Research Unit on Lipids and Atherosclerosis, “Sant Joan” University Hospital, Institut d’Investigació Sanitària Pere Virgili-CERCA, Universitat Rovira i Virgili, 43204 Reus, Spain; 7 Centro de Investigación Biomédica en Red de Diabetes y Enfermedades Metabólicas (CIBERDEM), 28029 Madrid, Spain; 8 Translational Regenerative Neurobiology Group, Molecular and Integrative Biosciences Research Program (MIBS), Faculty of Biological and Environmental Sciences, University of Helsinki, 00014 Helsinki, Finland

**Keywords:** collagen IV, branching, DDR1, MBP, oligodendrocytes, Olig2, phosphorylation, tyrosine kinase receptor

## Abstract

Discoidin domain receptor 1 (DDR1) is a tyrosine kinase receptor expressed in epithelial cells from different tissues in which collagen binding activates pleiotropic functions. In the brain, DDR1 is mainly expressed in oligodendrocytes (OLs), the function of which is unclear. Whether collagen can activate DDR1 in OLs has not been studied. Here, we assessed the expression of DDR1 during in vitro OL differentiation, including collagen IV incubation, and the capability of collagen IV to induce DDR1 phosphorylation. Experiments were performed using two in vitro models of OL differentiation: OLs derived from adult rat neural stem cells (NSCs) and the HOG16 human oligodendroglial cell line. Immunocytofluorescence, western blotting, and ELISA were performed to analyze these questions. The differentiation of OLs from NSCs was addressed using oligodendrocyte transcription factor 2 (Olig2) and myelin basic protein (MBP). In HOG16 OLs, collagen IV induced DDR1 phosphorylation through slow and sustained kinetics. In NSC-derived OLs, DDR1 was found in a high proportion of differentiating cells (MBP+/Olig2+), but its protein expression was decreased in later stages. The addition of collagen IV did not change the number of DDR1+/MBP+ cells but did accelerate OL branching. Here, we provide the first demonstration that collagen IV mediates the phosphorylation of DDR1 in HOG16 cells and that the in vitro co-expression of DDR1 and MBP is associated with accelerated branching during the differentiation of primary OLs.

## 1. Introduction

Myelination, which is the wrapping of axons with a membrane produced by oligodendrocytes (OLs) in the central nervous system (CNS) and Schwann cells in the peripheral nervous system (PNS), is a very active cellular process involving cell proliferation, differentiation, and movement [1]. OLs originate from CNS-resident oligodendrocyte progenitor cells (OPCs), whereas Schwann cells originate from neural crest precursor cells [2]. OLs and Schwann cells interact with extracellular matrix (ECM) molecules to orchestrate all these functions [3]. Collagen, an important, structural ECM molecule that activates myelination [4], utilizes as many as 46 different polypeptides to form 29 collagen types [5]. The most abundant types of collagens include fibrillar collagens, such as collagen types I–III, and network-forming collagens, such as collagen IV. Collagens are upregulated during nervous-tissue development and are involved in synaptogenesis, axon guidance, brain architecture, and myelination. In the adult brain, collagens are not expressed at high levels but are present in the basement membrane and in the environment surrounding neurons and glial cells [6,7]. The role of collagens in CNS myelination is not well understood. Conversely, accumulating evidence shows that collagens IV, V, VI, and XV affect multiple Schwann cell activities in the PNS such as adhesion, spreading, proliferation, differentiation, and myelination [8]. ECM collagens mediate these cellular and tissue functions mainly by binding to at least six different types of receptors, including integrins, adhesion G protein-coupled receptors (GPCRs), glycoprotein VI (GPVI), immunoglobulin-like receptor-1 (LAIR 1), OSCAR, and discoidin domain receptors (DDRs) [5]. Although GPVI, LAIR 1, and OSCAR are not expressed in the nervous system, integrins, GPCRs, and DDR1 are highly expressed in this system. GPR126, a member of the GPCR family expressed in the Schwann cell plasma membrane, binds collagen IV and plays an important role in PNS myelination [4]. Conversely, GPR56, which binds collagen III, is expressed in OLs and regulates cell proliferation in the CNS [9]. DDR1 is a tyrosine kinase receptor that utilizes native collagens as a ligand and exhibits unusually slow kinetics for kinase activity that lasts for hours or even days [10,11]. Type IV basement-membrane collagen is abundant in the brain and binds DDR1 but not DDR2 [10,11]. In turn, DDR1 mRNA is expressed at high levels in the developing and mature brain and exhibits a spatiotemporal expression pattern that is very similar to myelin basic protein (MBP) [12,13,14]. The DDR1 protein colocalizes with OL markers such as MBP and 2’,3’-cyclic nucleotide 3’-phosphodiesterase (CNP) in human brain tissue, human oligodendroglial cell lines, and mouse brain tissue [12,14,15]. Recent studies performing single-cell RNA sequencing (RNA-seq) analyses in the developing mouse brain showed that DDR1 mRNA expression peaks in the period characterized by newly formed OLs (NFOLs) and the beginning of myelinating OLs (MyOLs) [16]. However, the expression of DDR1 during primary OL differentiation has not been studied. Moreover, although studies utilizing different cell types have thoroughly described the collagen-induced activation of DDR1 [17,18], data on DDR1 activation in the brain are scarce. Bhatt et al. [19] showed that DDR1 and collagen IV colocalize at the pial surface in neurons in the developing mouse cerebellum. Although the expression of DDR1 in the myelin of the human adult CNS has been described [14], the ligand capable of activating the receptor in OLs remains unknown. The aims of this preliminary study are (1) to confirm that collagen IV is able to activate DDR1 in OLs using the oligodendroglial human cell line HOG16 as a model of MyOLs; (2) to explore the colocalization of the DDR1 and MBP proteins during the differentiation of primary OL cultures derived from rat neural stem cells (NSCs); and (3) to explore the morphological changes induced by collagen IV incubation during the differentiation of primary OL cultures. Here, we provide the first demonstration that collagen IV mediates the phosphorylation of DDR1 in HOG16 OLs and that the in vitro co-expression of DDR1 and MBP is associated with accelerated branching during the differentiation of primary OLs.

## 2. Results

### 2.1. Collagen IV Induces Y792-DDR1 Phosphorylation in HOG16 Oligodendroglial Cells

We conducted experiments using the human OL cell line HOG16 to determine whether collagen is able to activate DDR1 in myelinating cells in the brain. The rationale for the choice of this cell type was that this cell line is of human origin, produces myelin proteins such as MBP, MOBP, CNP, and DDR1 when incubated in a differentiation medium [15], and represents a homogenous population of “mature” OLs (the rat primary OL culture model used in this study is a heterogeneous cell population composed of OLs at different stages of differentiation [20]).

**Figure 1:**
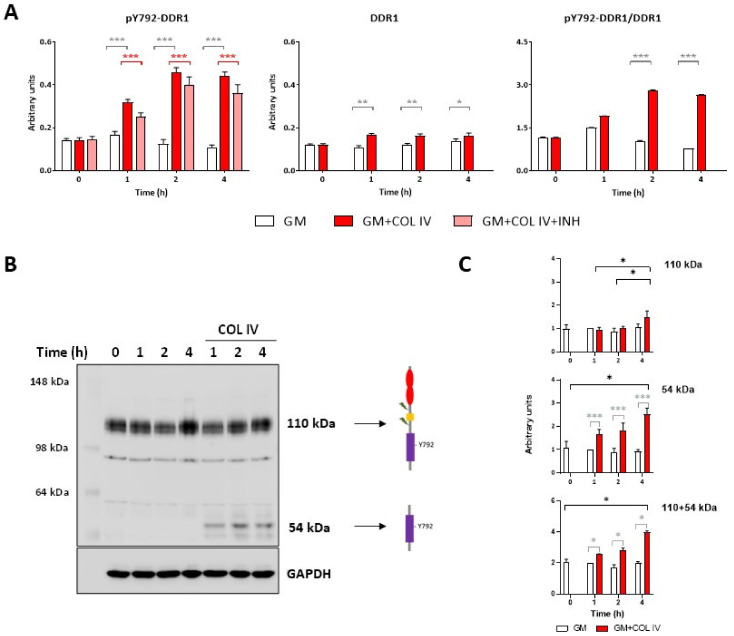
Measurement of the phosphorylated and total DDR1 levels in HOG16 cells incubated with collagen IV. (**A**) The levels of pY792-DDR1 and total DDR1 and the pY792-DDR1/DDR1 ratio (details in the Material and Methods section) in HOG16 cells incubated with growth medium (GM, white bars), GM plus 10 µg/mL collagen IV (COL IV, red bars), and GM plus 10 µg/mL collagen and 5 µM DDR1-IN-1 (pink bars) were measured by ELISAs. Higher pY792-DDR1 levels were detected at all time points in the cells cultured in the presence of collagen IV (with and without DDR1-IN-1). The pY-DDR1 level reached a plateau after 2 h of incubation with collagen, and the maximal pY792-DDR1/DDR1 ratio value was observed at 2 h. DDR1-IN-1 deceased the total DDR1 Y792 phosphorylation level by 20% at all time points. At 1, 2, and 4 h, the total DDR1 levels were higher in cells incubated with collagen than in cells incubated with GM. The time × treatment interaction was statistically significant for both DDR1 and pY792-DDR1, indicating that the treatment effect was different at each time point (Supplementary Table S1). The *p* values, determined by the one-way ANOVA at each time point to test the effect of treatment, are shown as gray lines (GM + COLIV compared with GM) and red lines (GM + COLIV + INH compared with GM + COLIV). (**B**) Representative western blotting showing the detection of phosphorylated DDR1 using anti-pY792 antibody (details in the Material and Methods section). (**C**) The relative absorbances of the 110- and 54-kDa bands was calculated from three different experiments and are shown individually (upper and middle chart) and summed (bottom chart). The relative absorbances of the bands showed an increase in the intensities of both the 110- and 54-kDa bands with increases in the incubation time with COL IV (the time × treatment interaction was statistically significant for both bands, indicating that the treatment effect was different at each time point; Supplementary Table S2). Notably, a significant increase in the 54-kDa band was observed at all time points after incubation with COL IV. The *p* values determined by the one-way ANOVA at each time point to test the effect of treatment are shown as gray lines * *p* < 0.001, ** *p* < 0.0001 and *** *p* < 0.00001.

Figure 1A shows the ELISA quantification of phosphorylated DDR1 (specifically at tyrosine 792, pY792) and total DDR1 in cells cultured in the presence or absence of 10 μg/mL of collagen IV. The presence of collagen IV in the incubation medium increased the level of pY792-DDR1 by threefold when compared with that in cells incubated in growth medium (GM) alone, and the maximum levels were observed after 2 h and were sustained for up to 4 h (the two-way ANOVA *p* values are shown in Supplementary Table S1, and the one-way ANOVA *p* values are shown in Figure 1A). Moreover, the addition of DDR1-IN-1, a specific DDR1 kinase inhibitor [21], to the incubation medium in the presence of collagen IV decreased DDR1 phosphorylation by 20% at all time points (Tukey’s test of the effects of time and treatment, shown in Supplementary Table S1). Interestingly, we observed a statistically significant increase in the level of DDR1 protein 1 h after the addition of collagen IV (a 1.5-fold increase compared to the baseline values), and this increase was sustained during the 4 h experiment (Figure 1A, a two-way ANOVA of the effect of time and treatment, Supplementary Table S1). A quantitative PCR showed that the addition of collagen IV increased the mRNA expression of the isoforms DDR1b (at 1, 2 and 4 h) and DDR1c after 4 h (Supplementary Figure S1). The ratio of pY792-DDR1 to total DDR1 (Figure 1A) was approximately three; it reached its maximum value after 2 h of incubation with collagen IV (Tukey’s test results are shown in Supplementary Table S1, and the one-way ANOVA *p* values are shown in Figure 1A). Notably, DDR1 phosphorylation was maintained for 24 h in the presence of collagen (Supplementary Figure S2). DDR1 phosphorylation was also measured by western blotting with an anti-pY792 antibody (the same antibody used in the ELISA). HOG16 cells constitutively present full-size receptor (110-kDa band) Y792 phosphorylation when incubated with differentiation medium (Figure 1B) without collagen (t = 0) and in regular growth medium (not supplemented with insulin, triiodothyronine, selenium, and transferrin with and without fetal bovine serum (FBS); Supplementary Figure S3), although to a lesser extent. Incubation with collagen IV increased the total receptor (110 + 54-kDa bands) phosphorylated intensity (Figure 1C and Supplementary Table S1). Interestingly, 2 and 4 h of incubation with collagen IV induced a marked and statistically significant increase in the 54-kDa phosphorylated band (Figure 1C and Supplementary Table S1). Two additional bands corresponding to a MW of 90 and 62 kDa without increased phosphorylation were observed after collagen incubation. HCT116 tumor cells, which were used as a positive control, showed a high expression of DDR1 compared with HOG16 cells, and incubation with collagen IV also increased the presence of the 54-kDa phosphorylated band. Based on these results, we conclude that collagen IV increases DDR1 phosphorylation, induces DDR1 cleavage, and thereby favors the formation of the 54-kDa fragment in HOG16 oligodendroglial cells.

### 2.2. DDR1 Is Temporally Expressed in Newly Formed OLs Derived from NSCs

**Figure 2:**
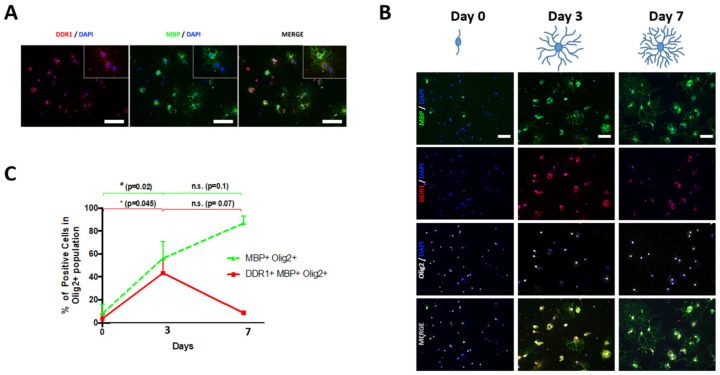
DDR1 is expressed during oligodendrogenic progression. Neural stem cells (NSCs) were obtained from the adult rat subventricular zone, expanded as neurospheres, and thereafter enzymatically dissociated and plated as individual cells. After exposure to pericyte-conditioned culture medium (PC-CM), NSCs differentiated into mature OLs (day 7). (**A**) Fluorescence images displaying DDR1 (red) and MBP (green) and DDR1-MBP colocalization (merge panel) in NSCs after x days of exposure to PC-CM. DAPI was used for nuclear counterstaining (blue). Scale bar = 50 μm. (**B**) Fluorescence images displaying DDR1+, MBP+, and Olig2+ cells after 0, 3, and 7 days of incubation with PC-CM. DAPI was used for nuclear counterstaining (blue). Scale bar = 20 μm. (**C**) Quantitative analysis showing MBP+ (green line) and DDR1+ and MBP+ (red line) cells among the total population of Olig2+ cells after 0, 3, and 7 days of exposure to PC-CM. The data show that the DDR1 levels peaked at day 3 and declined at day 7, whereas MBP expression continued to increase during oligodendrogenic progression. The data were obtained from three independent experiments and were analyzed by analysis of variance (ANOVA) followed by Tukey’s post hoc test. # and * *p* < 0.05, n.s.: not significant. * and # indicate significant differences between DDR1+ MBP+ cells and MBP+ cells, respectively, and the control group.

To test DDR1 protein expression during OL differentiation, we used a model of OL differentiation by exposing rat NSCs to an oligodendrogenic stimulus (pericyte conditioned medium, PC-CM [22]) for 0, 3, and 7 days. Under these conditions, cells can be identified as undifferentiated OPC-like (0 days) [20], newly formed OLs (3 days) and mature OLs (7 days) [22,23]. DDR1-expressing cell counting during OL differentiation (Olig2+ cells) was performed by immunocytofluorescence; the results are shown in Figure 2. First, we observed that DDR1 and MBP were co-expressed in the processes and in the soma in cultured OLs (Figure 2A). Second, we found that the maximal co-expression of DDR1 and MBP in Olig2+ cells occurred in newly formed OLs (day 3) (Figure 2B,C). Specifically, before application of the stimulus (day 0), only a few undifferentiated NSCs expressed DDR1 (4.36 ± 1.67%); subsequently, after 3 days of stimulation, a high percentage (40.15 ± 21.12%) of NSC-derived differentiating OLs (MBP+/Olig2+) expressed DDR1. Interestingly, DDR1 expression did not persist at later stages because very few mature OLs were positive for this receptor (8.45 ± 0.41%) after 7 days of stimulation, although the number of MBP+/Olig2+ cells continued to increase (Figure 2C).

### 2.3. Collagen IV Promotes Branching in Newly Formed OLs Derived from NSCs

**Figure 3:**
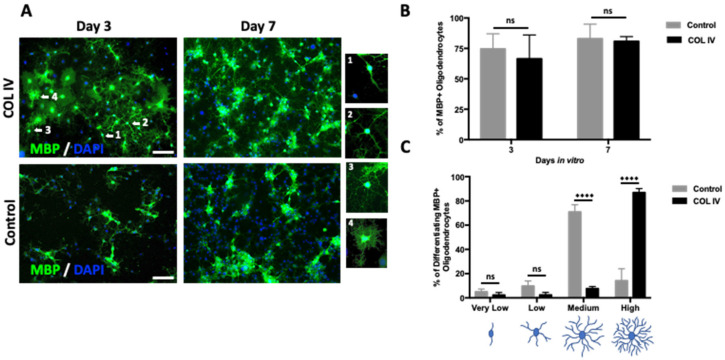
Collagen IV boosts OL maturation. The presence of COL IV accelerates the maturation of cultured NSCs to mature OLs. We identified four distinct morphologies (see bottom figure) of MBP+ cells, from immature OLs displaying primary processes with very low or low numbers of secondary branches to more mature multipolar OLs showing a medium or high degree of branching. (**A**) Representative fluorescence images displaying MBP+ (green) cells after 3 and 7 days of incubation under oligodendrogenic stimulus (control), supplemented or not supplemented with COL IV. DAPI was used for nuclear counterstaining (blue). Scale bar = 50 μm. Examples of four distinguishable MBP+ cells morphologies (arrows) are shown at high magnification with increasing complexity: (1) bipolar cells with a very low degree of branching; (2) multipolar cells with a low degree of branching; (3) multipolar cells with a medium degree of branching; and (4) multipolar, highly ramified cells. (**B**) Quantitative analysis showing the proportion of MBP+ cells among the total cell number after 3 and 7 days of incubation with COL IV and the control conditions. Note that no significant differences in MBP+ expression were found between the different conditions. (**C**) Quantitative analysis of the MBP+ cell morphology distribution after 3 days of incubation with COL IV and control conditions. The four morphologies were analyzed according to panel A. The data show that COL IV boosts OL maturation compared with that observed under control conditions. The data were obtained from three independent experiments and were analyzed by analysis of variance (ANOVA) followed by Bonferroni post hoc test. **** *p* < 0.0001, ns: not significant.

To investigate whether collagen IV influences DDR1 expression in MBP+/Olig2+ OLs, NSCs were exposed to oligodendrogenic conditions in the presence or absence of collagen IV. First, collagen IV did not alter the proportion of MBP+ cells among the total number of cells (Figure 3A,B) or the proportion of DDR1+ cells among MBP+/Olig2+ cells. Second, we observed a significant increase in the percentage of MBP+ OLs displaying a complex, multipolar, highly branched morphology after collagen IV exposure for 3 days (Figure 3A,C). Notably, this complex morphology, achieved by MBP+ OLs after 3 days of exposure to collagen IV (Figure 3C), is comparable to the highly arborized morphology observed after 7 days under control conditions (Figure 2B). These results indicate that the presence of collagen IV facilitates and accelerates OL maturation.

## 3. Discussion

Here, we provide the first demonstration that the incubation of OLs with collagen IV increases DDR1 phosphorylation and shedding, transiently increases DDR1 protein expression during OL lineage progression, and boosts OL-processes branching. DDR1, a protein that is expressed at high levels in brain OLs, is a transmembrane tyrosine kinase receptor whose only ligand described to date is collagen. However, the specific ligand that activates DDR1 in OLs has not been described, and the specific role of DDR1 in OLs has not been elucidated. A single-cell RNA-seq study confirmed that DDR1 expression in cells isolated from the mouse brain increases threefold during the transition of OPCs to NFOLs. DDR1 is the fourth-ranked gene in the list of the top 20 membrane receptors expressed and is co-expressed with classic myelin genes [24]. A co-expression network analysis of DDR1 during human brain development showed that DDR1 is co-expressed mainly with myelin and oligodendrocyte genes [13]. In a recent review, we highlighted the increase in DDR1 mRNA expression at the initiation of myelination [16]. Here, we provide the first demonstration of a transient increase in DDR1 protein expression during OL lineage progression. Although DDR1 is virtually absent in undifferentiated stem and progenitor cells, its expression increased during OL differentiation but decreased at later stages. Here, we found that collagen IV accelerates branching during maximal DDR1 expression. These preliminary findings suggest a functional role for DDR1 during OL development and eventually in early myelination, but this result requires further investigation. Moreover, collagen IV induced DDR1 phosphorylation in an oligodendroglial cell line (HOG16 cells) with slow kinetics: the phosphorylation peaked 2 h after the addition of collagen, and this peak was maintained 4 h after collagen addition. The same phosphorylation pattern has also been observed in other cell types [18,25]. In addition, the incubation of HOG16 cells with collagen IV produced a rapid (<1 h) and marked increase (1.5-fold) in the number of detectable DDR1 receptors. This rapid increase might indicate that collagen binding to cell surface receptors either accelerates the maturation of an intracellular storage pool of “immature” DDR1 receptors or favors receptor recycling, as has been described for other tyrosine kinase receptors [26]. In addition, mRNA expression data suggest that the addition of collagen IV induces an isoform-dependent increase with significant overexpression of the DDR1b and DDR1c isoforms. This result deserves future investigation. Notably, incubation with collagen IV substantially increased the amount of the phosphorylated, intracellular 54-kDa fragment. This result agrees with previous reports showing that collagen incubation enhances DDR1 cleavage [10,27,28,29]. Moreover, Fu et al. [17] observed that, in COS cells transfected with DDR1a, 62-kDa fragment phosphorylation occurs independently of collagen stimulation, which agrees with our finding of the presence of a constitutively phosphorylated 62-kDa band. Regarding the 90-kDa phosphorylated band (although it has not been described), it is interesting to note that a previous work [28] using the T47D cell line incubated with collagen I yielded a western blot (WB) band with a similar molecular weight. The nature of this band is difficult to interpret. Altogether, the results indicate the phosphorylation and production of an intracellular fragment (62 kDa) independent of collagen incubation and the production of a collagen-induced 54-kDa band. Regarding receptor intracellular fragments, it has been postulated that these fragments could be translocated to the nucleus and play a transcriptional regulatory role [16,17]; similarly, these fragments can bind downstream proteins different from those that bind to the full-length activated receptor [17]. The incubation of HOG16 cells without differentiation reagents and without 10% FBS still yielded the 110-kDa phosphorylated DDR1 band; therefore, we can assume that DDR1 is constitutively phosphorylated in HOG16 cells. Notably, under these conditions, incubation with 10% FBS increased the phosphorylation band intensity, which suggests that some components in FBS stimulate DDR1 phosphorylation. Furthermore, HOG16 cells incubated with GM containing insulin, transferrin, triiodothyronine, and selenium also exhibited phosphorylated DDR1, even without collagen addition. According to these results, the insulin-induced phosphorylation of DDR1 has been described in cancer cells [30], and the constitutive phosphorylation of DDR1 is present in several oral squamous cell carcinoma cells [31] but not in other cell types, such as breast cancer T24D cells [10].

Using DDR1-IN-1, a selective DDR1 inhibitor that binds the hinge region of the receptor (aa 672, 702, 704 and 782), we observed a 20% reduction in Y792 phosphorylation. Our results from HOG16 OL cells contrast with previous findings showing that the inhibitor produced a greater reduction in overall receptor phosphorylation [21,32]. In part, we did not expect a greater reduction in phosphorylation because this inhibitor mainly targets Tyr703 [21] and the intracellular domain of DDR1 contains 15 Tyr residues that can be phosphorylated; the commercially available ELISA kit that we used recognizes phosphorylation at Tyr792 [33]. Moreover, as was observed in previous studies, the expression of DDR1 is increased in the osteosarcoma cell line U2OS after incubation with 2 µg/mL of doxycycline for 48 h [21,32]. At this concentration, doxycycline functions as an inhibitor of membrane-type metalloproteinase (MT-MMP), which, in turn, maintains higher levels of full-length DDR1 by preventing cleavage. The authors of the same article claimed that DDR1-IN-1 is an incomplete inhibitor of DDR1-mediated signaling that perhaps acts on other kinases because it is selective for DDR1 but does not inhibit subsequent cell proliferation like other inhibitors [21]. However, we are unable to exclude the possibility that the inhibitor might bind to other tyrosine kinases in our experiments and thus influence the DDR1 phosphorylation. Altogether, these results suggest that the phosphorylation induced by collagen IV, at least at Y792, is partially blocked by the DDR1-IN-1-specific inhibitor, but further investigations are warranted.

In the PNS, the myelin sheath is provided by Schwann cells. Collagen VI is important for PNS myelination [34]. Collagen present in the ECM of the PNS and CNS might, together with other molecules, provide guidance for oligodendrocytes during neurodevelopment and remyelination. In fact, GPR126 [9] in Schwann cells and GP56 in oligodendrocytes [4] bind collagen IV and III, respectively, and are related to myelination [4,9,35]. Here, we found that the incubation of NSC-derived OLs with collagen IV accelerates OL branching, which can be interpreted as OL maturation. Although we did not demonstrate through which OL receptor collagen IV accelerates cell maturation, we can postulate that integrins, GPRs, and DDR1 probably orchestrate the interaction with ECM collagens and contribute to the complex process of Schwann cell/OL lineage differentiation [8]. Furthermore, it is well known that DDR1 intracellularly binds non-muscle myosin IIA [36,37] and mediates extracellular collagen contraction [38]. All these results support the hypothesis that DDR1, upon binding to collagen, participates in the OL cell morphology changes associated with cell maturation, as has been demonstrated for other cell types [5]. Future experiments designed to test this hypothesis are needed.

Compelling evidence shows that abnormalities in CNS myelination are the cause of certain leukodystrophies [39] present in patients with multiple sclerosis [40] and which contribute to schizophrenia pathology [41]. DDR1 is upregulated in OLs during neurodevelopment, myelination [12], and remyelination [15]. Additionally, we identified an association between DDR1 genetic variants and schizophrenia [42,43]. In a subsequent study, we observed higher levels of DDR1c in brain tissue derived from patients with schizophrenia when compared to healthy controls [44]. According to a recent study, patients with schizophrenia who are carriers of the risk alleles for the DDR1 variant rs1264323 exhibit lower myelin integrity in brain regions associated with the cognitive processing speed [42]. Moreover, mutations in DDR1 were found to be associated with 11% of Schwannoma-cell tumors in a recent study of a large series of patients with Schwannoma [45]. In addition, DDR1 is upregulated in human gliomas [46]. Therefore, alterations in DDR1 expression may lead to CNS myelin instability/malfunctions that contribute to pathology, but this hypothesis requires further examination.

Some limitations of the present study must be mentioned. First, the experiments described in the present study were performed using HOG16 oligodendroglial cells, a cell line with features of OLs and characteristics of transformed cells [47,48]. Nevertheless, OLs derived from adult NSCs co-expressed DDR1 and MBP, which suggests that our findings may also be valid for primary cultures. However, further in vivo studies aiming to address the role of DDR1 in CNS-resident myelinating cells are needed to reveal its physiological function. Second, in HOG16 cell cultures, collagen derived from FBS was removed before all the experiments. Thus, the cells were incubated for at least 18 h in GM without FBS, and although the GM contained essential elements for the survival of HOG16 cells, including insulin, selenium, triiodothyronine, and transferrin, the fact that many other molecules in FBS may be required must be considered. Third, the experiments performed in the present study were conducted with the most abundant ECM collagen in the brain, collagen IV, but other collagen types that may have important functions in orchestrating myelination were not tested. Fourth, we used a commercially available antibody that detects phosphorylation at Tyr792 in the ELISA and WB experiments, and future studies using a broader set of reagents are needed to elucidate which Tyr residues are involved in DDR1 activation in OLs.

In summary, these results are relevant because they confirm that DDR1 is activated by collagen in OLs and that DDR1 is co-expressed with MBP during the OL lineage maturation process, which is accelerated by the presence of collagen. Based on these results, we postulate that DDR1 may play a role in myelination mediated by collagen.

## 4. Materials and Methods

### 4.1. HOG16 Cell Line and Culture

The human oligodendroglial cell line HOG16 was obtained from Eucellbank (Department of Cellular Biology, University of Barcelona, Barcelona, Spain) with the permission of Dr. G. Dawson (University of Chicago, Chicago, IL, USA). The cells were free of mycoplasma, determined by testing with commercial reagents (EZ-PCR Mycoplasma Test Kit, Biological Industries, Kibbutz Beit Haemek, Israel). The cell line was cultured in high-glucose DMEM (Gibco, Thermo Fisher Scientific Slu., Alcobendas, Spain) supplemented with 10% fetal bovine serum (FBS) (Gibco), 50 U/mL of penicillin, and 50 μg/mL of streptomycin (Gibco) in a 37 °C incubator with a humidified atmosphere of 5% CO_2_. HOG16 cells were cultured with differentiation medium (DM) containing 0.05% FBS, 30 nM of triiodothyronine, 30 nM of selenium, 0.5 μg/mL of insulin, 50 μg/mL of transferrin (all from Sigma-Aldrich, Madrid, Spain), and the antibiotics listed above for 48 h to induce differentiation. HOG16 cells cultured with DM exhibit morphological changes and an increased expression of myelin proteins such as MBP and myelin-associated oligodendrocyte basic protein (MOBP) [15,48,49]. Cells that grew in DM and reached 80% confluence were detached with EDTA-trypsin and seeded in six-well plates (70,000 cells/well). After 48 h of incubation, the DM was removed and the cells were incubated for 18 h in a FBS-free medium to avoid the presence of any collagen and other possible DDR1 ligands (referred to as growth medium, GM, throughout the manuscript for clarity). First, we tested different collagen and DDR1 inhibitor concentrations to select suitable concentrations for the experiments using HOG16 cells. We selected collagen IV (C6745, Sigma-Aldrich) because it exclusively activates DDR1 [10,11] and is the most abundant collagen in the basement membrane [5]. Collagen IV, which was dissolved in 0.25% acetic acid, was tested at final concentrations of 5, 10, and 20 μg/mL and was ultimately used at a concentration of 10 μg/mL. The DDR1-IN-1 inhibitor (kindly donated by Dr. Jinhua Wang, Dana Farber Cancer Institute, Boston, MA, USA) [21] was selected as a very selective inhibitor of DDR1 autophosphorylation. DDR1-IN-1, which was dissolved in water, was tested at final concentrations of 1, 5, and 10 μM. According to Kim et al. [21], at a final concentration >10 μM, the inhibitor interacts with other kinases. In our hands, the inhibitor concentration of 5 μM decreased DDR1 phosphorylation by 10% when compared with that obtained with 1 μM. Therefore, we used a final concentration of 5 μM in the cell medium. HCT116, a tumor cell line that highly expresses DDR1, was used as a positive control in the western blot experiments as previously described [14]. All experiments were performed in triplicate, and a time-course experiment was performed with the time points of 1, 2, and 4 h. In some experiments, an overnight incubation was also included. Triplicate wells were incubated under each different treatment condition in each of the three experiments.

### 4.2. Adult Neural Stem Cell Cultures

Rat subventricular zone (SVZ) NSCs were prepared as previously described [50]. Briefly, 2-month-old female Fisher 344 rats were anesthetized with isoflurane and subsequently decapitated. SVZs were dissected and collected in ice-cold Dulbecco’s phosphate-buffered saline (PBS) containing glutamine (DPBS/glu). The brain tissue was minced, enzymatically digested, washed, and cultured in an NBA medium (Gibco, Thermo Fisher Scientific, Waltham, MA, USA) containing epidermal growth factor (EGF) and fibroblast growth factor 2 (FGF2) to induce neurosphere formation. For in vitro expansion, NSCs were cultured in T25 flasks in a humidified incubator (20% O_2_ and 5% CO_2_ at 37 °C).

### 4.3. Primary OL Cultures Derived from Adult Rat NSCs

OLs were prepared from NSCs as previously described [22]. Briefly, neurospheres were dissociated with Accutase, and NSCs were seeded onto polyornithine (250 µg/mL)-and laminin (5 µg/mL)-coated glass coverslips at a density of 50,000 cells/cm^2^ in Dulbecco’s Modified Eagle’s Medium (DMEM), supplemented with 20% Knockout Serum (Gibco) and cultured overnight. Subsequently, the medium was replaced, and the cells were incubated with a CNS pericyte-conditioned medium (PC-CM) [22]. PC-CM, supplemented with collagen IV, was dissolved in 0.25% acetic acid to a final concentration of 10 µg/mL, and the day in which the collagen was dissolved in the solvent was defined as day 0. On days 0, 3, and 7, the cells were fixed with 4% paraformaldehyde in PBS (Sigma-Aldrich, Darmstadt, Germany) for 10 min and processed for immunofluorescence staining.

### 4.4. ELISA

We used two ELISA kits (the PathScan Total DDR1 Sandwich ELISA kit and the PathScan PhosphoDDR1 Sandwich ELISA kit, Cell Signaling Technology, Inc., Beverly, MA, USA) to quantify the total and phosphorylated DDR1 (DDR1 and pY-DDR1, respectively) levels in HOG16 cells. After incubation with GM and collagen in the presence or absence of the DDR1-IN-1 inhibitor, the cells were washed with 1× PBS and lysed with lysis buffer (Cell Signaling Technology, Inc.) for 5 min on ice. The cells were collected with scrapers and centrifuged at 14,000 rpm for 10 min at 4 °C. The lysates were immediately frozen at −80 °C and stored until use. Prior to ELISA, the protein concentration was measured with a Qubit fluorimeter (Life Technologies, Madrid, Spain) to normalize the protein quantities in each well. The total DDR1 ELISA kit uses wells coated with a rabbit polyclonal Ab against human DDR1 and a mouse monoclonal Ab against the extracellular domain (N-terminus) of human DDR1 to detect the bound receptor. The phosphoDDR1 kit uses wells coated with a rabbit Ab against human DDR1 to capture the receptor and a mouse monoclonal Ab is used against phosphorylated human DDR1 (Tyr 792) to detect receptor phosphorylation. The ELISAs were performed according to the manufacturer’s instructions. Each condition of the biological experiment was analyzed in triplicate ELISA plate wells, and a Synergy HT ELISA plate reader (BioTek Instruments, Inc. Winooski, VT, USA) was used to measure the absorbance of each well. The data are presented as relative absorbance units. Outlier values (>±2 SD) were excluded from the analysis.

### 4.5. RT-qPCR

HOG16 cells were plated on six-well plates and cultured under the appropriate experimental conditions (see the HOG16 cell line and culture section). After incubation with GM or GM supplemented with collagen IV, the cultures were scraped, and the cell pellets were resuspended in cold 1× PBS to obtain a final dilution of 20,000 cells/μL. The TaqMan Gene Expression Cells-to-Ct^TM^ kit (Invitrogen, Life Technologies, Madrid, Spain) was then used to directly analyze the mRNA expression levels in the cultured adherent cells by following the manufacturer’s instructions. For the qPCR assay, DDR1 Assays-on-Demand Gene Expression assays and three custom TaqMan assays (Invitrogen, Life Technologies) were used to detect the expression of the DDR1a, DDR1b, and DDR1c isoforms [49]. The qPCR amplification of HOG16 cells was performed in triplicate for each target gene, and each biological experimental condition in a final reaction volume of 10 μL containing 2 μL of cDNA, 5 μL of TaqMan Gene Expression Master Mix, 0.5 μL of TaqMan Gene Expression Assay buffer, and nuclease-free water (Life Technologies). We quantified the relative mRNA expression levels of total DDR1 and the DDR1a, DDR1b, and DDR1c isoforms using RPLP0 and β2 M as the reference genes [49]. The qPCR data were acquired using the 7900HT Sequencer Detection System (SDS version 2.3; Life Technologies) and analyzed using Expression Suite (Version 1.2) software with the 2^−ΔCq^ method (Invitrogen, Life Technologies). Samples with a coefficient of variation >3% were excluded.

### 4.6. Western Blot

We performed western blotting to confirm the phosphorylation of DDR1 in cells incubated with collagen IV. Briefly, HOG16 cells were lysed with a Laemmli sample buffer (300 µL/10^6^ cells), and 15 µL of HOG16 cell lysates was separated by electrophoresis on 8% Tris-glycine gels under reducing conditions at 160 V for 1 h. The proteins were then transferred to nitrocellulose membranes using an iBlot 2 Gel Transfer Device (Thermo Fisher Scientific IB21001), blocked with 4% skim milk and 0.1% Tween-20 in 1× Tris-buffered saline (TBS) for 1 h at room temperature (RT), and then incubated overnight with the primary antibodies. Phosphorylated DDR1 was detected with a rabbit anti-Tyr792 DDR1 antibody (#11994, Cell Signaling Technology, Leiden, The Netherlands) at 1:500 dilution. Notably, this is the same antibody used in the ELISA. Additionally, DDR1 was detected with an antibody against the C-terminal intracellular fragment (sc-374618, Santa Cruz Biotechnology, Santa Barbara, CA, USA) at 1:100 dilution. This was followed by incubation with the reagent DDR1 m-IgGκ BP-HRP (Santa Cruz Biotechnology, sc-516102) at 1:1000 dilution. A rabbit anti-GAPDH antibody (#5174, Cell Signaling Technology, Leiden, The Netherlands) at 1:1000 dilution served as a loading control. The membranes were then incubated with a goat peroxidase-conjugated anti-rabbit antibody (#P0448, DAKO, Glostrup, Denmark) diluted 1:10,000. All secondary antibodies were incubated for 1 h at RT. Antibody binding was detected using ECL™ Select Western Blotting Detection Reagent (#RPN2235, Sigma) and recorded using the Amersham Imager600 molecular imaging camera (GE Healthcare, Barcelona, Spain). The molecular weights of the bands were assigned by comparing the band weights with the SeeBlue™ Plus2 Prestained Protein Standard (LC5925, Invitrogen). Densitometry analysis of the captured images was performed using ImageQuant TL software, version 8.1 (GE Healthcare, Barcelona, Spain). After subtracting the background value, each band was normalized to the GAPDH band and calculated relative to that for the 1-h incubation condition without collagen, which was set to 1. The results are presented as relative units.

### 4.7. Immunocytofluorescence

Fixed NSCs were washed with PBS and then blocked with a solution composed of PBS, 0.1% Triton-X100 (only for intracellular antigens), 1% bovine serum albumin (BSA) and 0.2% teleostean gelatin (Sigma-Aldrich, Darmstadt, Germany). The same solution was used for incubation with antibodies. The cells were incubated with the primary antibodies overnight at 4 °C. Fluorochrome-conjugated, species-specific secondary antibodies were used for immunodetection. The following primary antibodies and final dilutions were used: mouse mAb anti-C-terminus (intracellular domain) DDR1 antibody (C6, #sc-374618, Santa Cruz Biotechnology, Dallas, TX, USA) diluted 1:50, rat polyclonal Ab against MBP (AbD Serotec, Kidlington, UK) diluted 1:500, and rabbit polyclonal Ab against Olig2 diluted 1:500 (AB9610, Sigma-Aldrich, Darmstadt, Germany). The following secondary antibodies were used: donkey anti-rat, donkey anti-mouse, and donkey anti-rabbit conjugated with Alexa Fluor 488, Alexa Fluor 568, and Alexa Fluor 647, respectively, all diluted 1:500 (Invitrogen, Thermo Fisher Scientific, Waltham, MA, USA).

Fixed HOG16 cells were permeabilized with 0.1% BSA in 1× PBS containing 0.5% Triton X-100 for 5 min at 20 °C and rinsed again with 1× PBS. The cells were then incubated overnight at 4 °C with the primary anti-C-terminus DDR1 (intracellular domain) antibody (1:100 dilution, SC-532; Santa Cruz Biotechnology, Madrid, Spain). The slides were rinsed and incubated for 3 h at 37 °C with the appropriate fluorochrome-conjugated secondary antibody (Jackson ImmunoResearch, West Grove, PA, USA). Nuclear counterstaining was performed with 0.25 μg/μL of 4’,6’-diamidino-2-phenylindole dihydrochloride hydrate (DAPI; Sigma, Darmstadt, Germany). The specimens were mounted on microscope slides using Dako Fluorescence Mounting Medium (Dako, Denmark). Epifluorescence observations and photodocumentation were performed using a LEICA DM 2000 LED microscope (Leica, Munich, Germany) equipped with a digital camera and Ocular^TM^ software version 2.0.1.496 (QImaging, Surrey, BC, Canada) for NSCs. A Nikon confocal microscope (TE2000-E) with EZ-C1 Nikon software and MacBiophotonics ImageJ 1.43 m software were used for processing the data obtained from HOG16 cells.

### 4.8. Statistical Analyses

A two-way analysis of variance (ANOVA) was used to measure the effect of time or treatment alone or time and treatment on the mean DDR1 levels obtained by ELISA and immunocytofluorescence. In addition, one-way and two-way ANOVAs were used to compare treatment conditions at a certain time point. Tukey’s and Bonferroni post hoc tests were used to identify differences between treatment conditions and time points in each experiment. Statistical significance was set to a *p* value < 0.05 in each experiment. The statistical analyses were conducted using IBM SPSS Statistics for Windows, version 20.0 (IBM Corp., Armonk, NY, USA) and GraphPad Prism 9.4.1 software (GraphPad Software, La Jolla, CA, USA)

## Data Availability

The data presented in this study are available in the article and supplementary materials.

## Supplementary Materials

The following are available online at https://www.mdpi.com/article/10.3390/ijms24021742/s1.
