# Supplementary material for: DDR1 and Its Ligand, Collagen IV, Are Involved in In Vitro Oligodendrocyte Maturation
Source: Int J Mol Sci. 2023 Jan 16;24(2):1742. doi: 10.3390/ijms24021742 (PMC9866737; doi:10.3390/ijms24021742)
Supplement: Supplementary file 1 [file ijms-24-01742-s001.zip › ijms-1993944-supplementary.pdf]

Supplementary Table S1. Two-way ANOVA comparing total DDR1, pY DDR1 and pY DDR1/DDR1 measured by ELISA using time and treatment as fixed factors.

	DDR1				pY DDR1				pY DDR1/DDR1			
**Factor**	**F**	**df**	**P**	**R^2**	**F**	**df**	**P**	**R^2**	**F**	**df**	**P**	**R^2**
Time	16.1	3	1.4x10^{-6}		437.3	3	4.8x10^{-26}		56.9	3	6.2x10^{-13}	
Treatment	22.2	3	5.2x10^{-8}	0.72	1330.9	3	1.2 x10^{-33}	0.99	227.1	3	1.2x10^{-21}	0.97
Time x treatment	2.8	9	1.3x10^{-2}		210.5	9	7.0x10^{-26}		56.8	9	3.8x10^{-17}	

Tukey post hoc test

**Factor**	**groups**	**P**	**groups**	**P**	**groups**	**P**
Time	0 vs 2	2.0x10^{-4}	0 vs 1	6.9x10^{-13}	0 vs 1	3.6x10^{-8}
	0 vs 4	1.1x10^{-6}	0 vs 2	6.9x10^{-13}	0 vs 2	9.8x10^{-13}
	1 vs 4	2.5x10^{-3}	0 vs 4	6.9x10^{-13}	0 vs 4	1.7x10^{-9}
			1 vs 2	7.0x10^{-13}	1 vs 2	1.7x10^{-4}
			1 vs 4	1.2x10^{-10}	2 vs 4	4.2x10^{-3}
			2 vs 4	3.9x10^{-4}		
Treatment	GM vs GM+COL	3.9x10^{-8}	GM vs GM+COL	6.9x10^{-13}	GM vs GM+COL	6.9x10^{-13}
	GM vs GM+COL+INH	6.9x10^{-6}	GM vs GM+COL+INH	6.9x10^{-13}	GM vs GM+COL+INH	7.0x10^{-13}
			GM+COL vs GM+COL+INH	9.4x10^{-13}	GM+COL vs GM+COL+INH	3.6x10^{-4}

Supplementary Table S2. Two-way ANOVA comparing pY792 DDR1 measured by WB using time and treatment as fixed factors.

	pY792 DDR1 (110+54 kDa)				pY792 DDR1-110 kDa				pY792 DDR1-54 kDa			
**Factor**	**F**	**df**	**P**	**R^2**	**F**	**df**	**P**	**R^2**	**F**	**df**	**P**	**R^2**
Time	3.10	3	0.056		4.16	3	0.023		7.04	3	4.0×10^{-3}	
Treatment	7.03	1	0.017	0.457	1.07	1	NS	0.352	62.84	3	1.5×10^{-6}	0.821
Time x treatment	3.34	3	0.046		1.97	3	NS		10.82	9	6.9×10^{-4}	
					**Tukey post hoc test**							
**Factor**		**groups**		**P**		**groups**		**P**		**groups**		**P**
Time		1 vs 4		0.055		1 vs 4		0.035		0 vs 4		0.016
						2 vs 4		0.040				

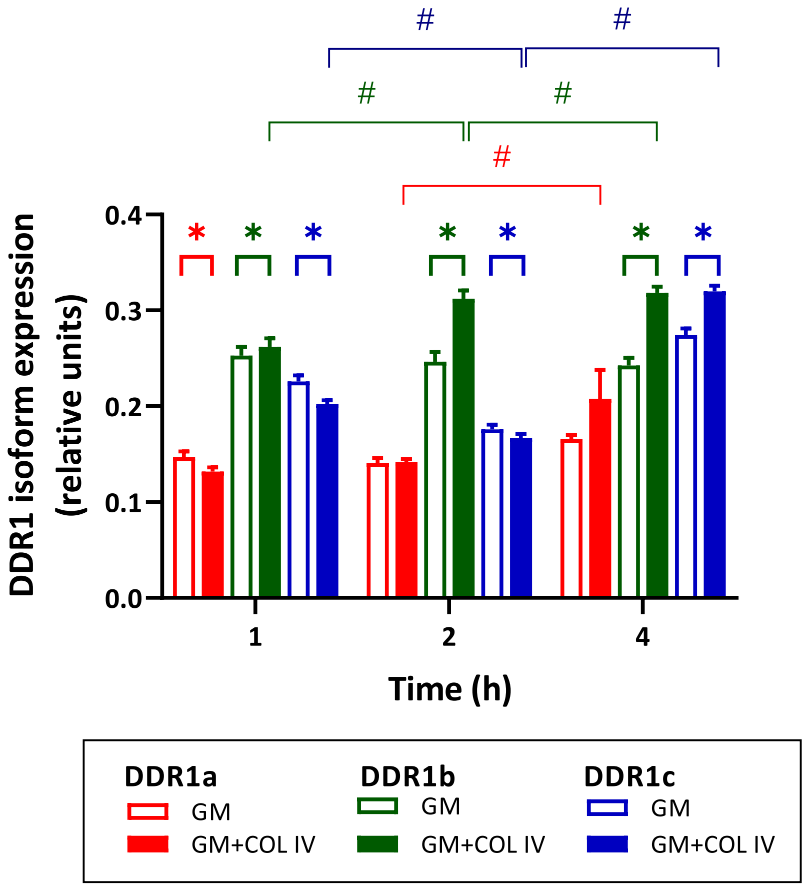

Figure S1. DDR1 isoform expression measured by RT-qPCR in HOG16 cells incubated in the presence of collagen IV.
HOG16 cell were incubated with GM with and without collagen IV for 0, 1, 2 and 4h. Total DDR1 and DDR1a, DDR1b and DDR1c isoform expression was determined by RT-qPCR as described in the Materials and methods section in the cell lysates. Relative expression is shown as DDR1 isoform expression ($2^{-\Delta cq}$)/total DDR1 expression ($2^{-\Delta cq}$). Results are the mean of 3 different experiments (+SEM). ANOVA (with Tuckey post hoc test, $p<0.05$) showed that collagen IV incubation altered the expression of DDR1b and DDR1c at all time points and only at 1h for DDR1a. To note that DDR1c expression decreased in the presence of collagen IV at 1 and 2h, but increased at 4h of incubation. DDR1b expression increased with the presence of collagen IV at all time points. A significant increase in the expression of all three isoforms was observed after 4h of incubation. Significant differences between GM and GM+COL IV are shown with *; and between time points differences are shown with #.

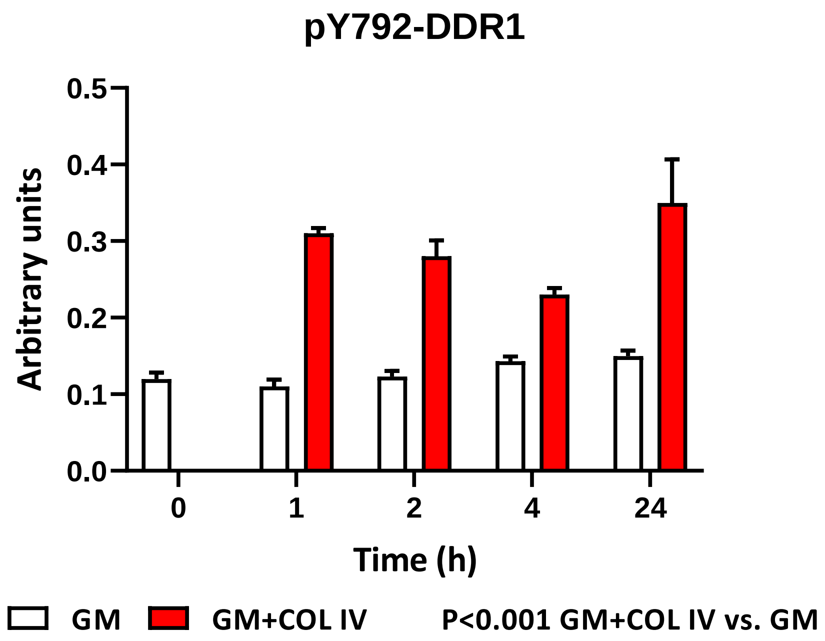

Figure S2. Phosphorylated DDR1 determined by ELISA in HOG16 cells in the presence of collagen IV for up to 24h. HOG16 cells were incubated with GM with and without collagen IV for up to 24h. pY792-DDR1 was detected by ELISA in the cell lysates as described in the Materials and methods section. Results are the mean of 3 different experiments (+SEM). ANOVA (with Tuckey post hoc test, $p<0.001$) showed that collagen IV incubation increased the level of pY792-DDR1 at all time points (from 1h up to 24h).

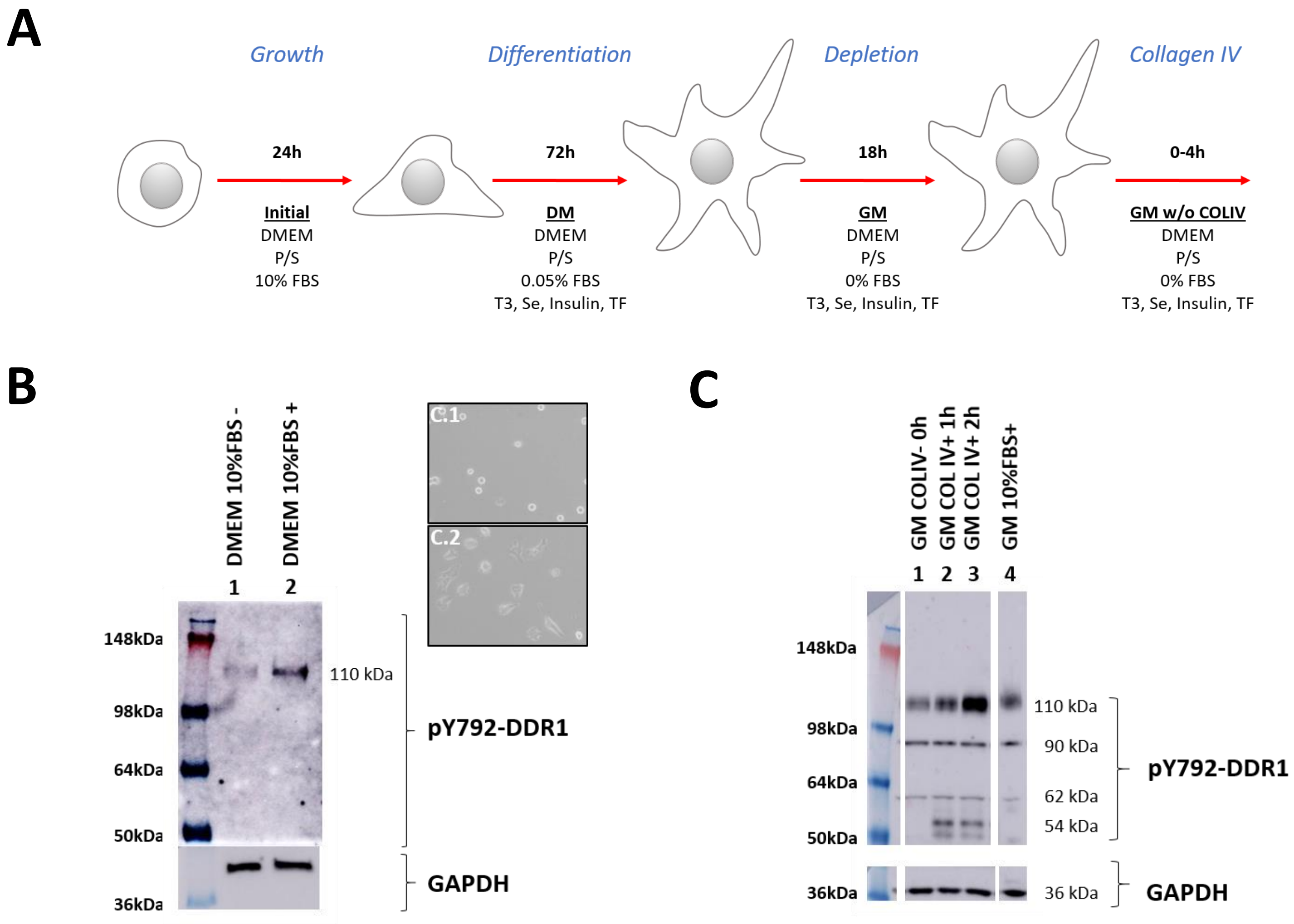

Figure S3. Western blotting measurement of phosphorylated DDR1 levels in HOG16 cells incubated with different growth medium.
A. Schematic representation of HOG16 cell culture from defrosting until collagen IV experiments. For methodological details see the Material and methods section. **B.** Non-differentiated HOG16 cells cultured with high-glucose DMEM without FBS (lane 1 and insert C.1) and with 10% FBS (lane 2 and insert B.2) for 12h. Under such conditions (without differentiation and 12h of incubation) cells were still floating in absence of FBS (C.1) and already attached when supplemented with 10% FBS, but in both cases the presence of pY792-DDR1 110 kDa band was detected. **C.** Differentiated and FBS-depleted HOG16 cells incubated with GM without FBS and without collagen IV (lane 1), with collagen IV for 1h (lane 2) and 2 h (lane 3). HOG16 cells non differentiated were incubated with DM supplemented with 10%FBS for 18h (lane 4). Note that handmade gels were used in B and commercial gels were used in **C**.
